# Aus Corona für die Zukunft familienfördernder Angebote lernen: Eine Fokusgruppenstudie mit Eltern und Fachkräften

**DOI:** 10.1007/s11553-022-00942-4

**Published:** 2022-04-11

**Authors:** Sonja Bröning, Annette Clüver

**Affiliations:** Fakultät Humanwissenschaften, Medical School Hamburg, Am Kaiserkai 1, 20457 Hamburg, Deutschland

**Keywords:** COVID-19, Familienförderung, Familienhilfe, Digitalisierung in der Sozialen Arbeit, Fokusgruppen, COVID-19, Family-based prevention, Family support, Digital social work, Focus group

## Abstract

**Hintergrund:**

Das Auftreten der Coronapandemie brachte für Familien mit kleinen Kindern vielfältige Belastungen wie Isolation, den Verlust öffentlicher und privater Betreuungsoptionen, sowie die Balance von Kinderbetreuung und Beruf. Zukunftssorgen, Verunsicherung und Gefühle der Einsamkeit trugen zum vermehrten Auftreten psychischer Probleme bei. Auch familienfördernde Einrichtungen standen vor neuen Herausforderungen: der Unterstützungsbedarf der Familien stieg, gleichzeitig waren bisherige Angebotsformate und Kontaktmöglichkeiten unter den Einschränkungsmaßnahmen schwer realisierbar.

**Ziel der Arbeit:**

Ziel der aktuellen Studie war die Erfassung der veränderten Bedarfslage von Familien im Verlauf der Pandemie und der Erfahrungswerte mit neu entstandenen analogen und digitalen Hilfsangeboten am Beispiel der familienfördernden Angebote der Stadt Hamburg.

**Material und Methoden:**

Fachkräfte unterschiedlicher familienfördernder Einrichtungen sowie deren Angebote nutzende Eltern wurden im Rahmen von Fokusgruppendiskussionen zu ihren Erfahrungen in der Pandemie und ihren zukunftsgerichteten Ideen für die Familienförderung befragt.

**Ergebnisse:**

Die Ergebnisse bestätigen die erhöhte Bedarfslage der Familien. Die Niedrigschwelligkeit der Angebote und der persönliche Austausch wurden von Familien und Fachkräften vermisst. Kreative Ansätze, Angebote digital zu ergänzen, wurden erprobt und z. T. als bereichernd wahrgenommen, besonders um neue Zielgruppen zu erreichen und interprofessionelle Kooperation unter Fachkräften zu stärken.

**Schlussfolgerung:**

Unterstützungsangebote in Präsenz müssen beibehalten werden, digitale Angebote bieten aber eine sinnvolle Ergänzung. Eine gelungene Kombination analoger und digitaler Angebote braucht effektive Ressourcenverteilung und Qualifizierung der Fachkräfte.

## Abstract (en)

**Background:**

The onset of the coronavirus pandemic created diverse stressors for families with small children such as isolation, limited public and private childcare options, and balancing work and childcare. Fear of the future, feelings of uncertainty and loneliness led to a rise in mental health problems. Public family support services also faced significant challenges: while families felt more need for support, established means of reaching families and providing services were severely curtailed.

**Objectives:**

The current study aimed at capturing pandemic-related changes in family needs and at documenting experiences in the use of newly developed analog and digital services provided by public family support institutions in the city of Hamburg, Germany.

**Materials and methods:**

We conducted focus groups with staff members of different types of public family support services and parents who were using these services. Central topics of discussion were pandemic-related experiences and ideas for the future of public family support services.

**Results:**

Results confirm an increase in family pressures. Parents and staff members missed low-threshold accessibility of services and personal contact and dialogue. Creative approaches to complement services digitally were developed. Several of these approaches were considered beneficial, especially for reaching new target groups and strengthening interprofessional cooperation in the field.

**Conclusions:**

In-person support services need to be upheld. However, digital services can effectively complement analog formats. A successful combination requires effective resource distribution and staff member qualification measures.

## Hinführung zum Thema

Die Coronapandemie bestimmte in den Jahren 2020/2021 den Alltag. Durch die Schließungen von Kitas und den Wegfall von Betreuungsoptionen waren Eltern einer Mehrbelastung ausgesetzt [2]. Diese war in Familien mit kleinen Kindern und geringen Ressourcen besonders hoch [5, 10]. Familienfördernde Angebote konnten häufig nicht in alter Form Entlastung schaffen [2, 8]. Daraus entstanden aber auch kreative, alternative Angebote. Erfahrungen hierzu aus Sicht von Eltern und Fachkräften werden in der vorliegenden Studie qualitativ erfasst und mit Blick auf mögliche Erkenntnisse für die Zukunft diskutiert.

## Hintergrund

Die Einschränkungen zur Eindämmung der Coronapandemie stellten für Familien einen massiven Eingriff in den familiären Alltag dar: Bildungs- und Betreuungsangebote für die Kinder standen für die meisten Familien nicht zur Verfügung und mussten von den Eltern selbst übernommen werden. Anderweitige Bezugspersonen und private Netzwerke konnten aufgrund der Kontaktbeschränkungen nicht zur Entlastung beitragen. Dazu kamen Vereinbarkeitsprobleme von Familie und Beruf, Wegfall sozialräumlicher Ressourcen wie Spielplätze oder Treffpunkte, psychische Probleme durch Isolation und Ängste sowie erhöhtes familiäres Konfliktpotenzial durch die Einengung auf den Wohnraum. Die psychische Mehrbelastung von Kindern und Jugendlichen durch die Pandemie ist bereits evident [11], was die Gefahr für Kindeswohlgefährdung im häuslichen Setting erhöht [12].

Der erhöhte Beratungs- und Unterstützungsbedarf der Familien stand im Kontrast zu wegfallenden Angeboten familienfördernder Einrichtungen (Familienbildung und -beratung, Erziehungsberatung, Frühe Hilfen) durch den „Lockdown“, sodass neue (zunächst kurzfristig organisierte) Formen der Hilfe entwickelt werden mussten. Dazu zählten sowohl analoge als auch digitale Angebote, wie z. B. eine verstärkte Telefonberatung, Beratungsspaziergänge, Nutzung von sozialen Medien. Da einheitliche Empfehlungen zur technischen Umsetzung, zum Datenschutz, zur Qualifizierung von Fachkräften und zu hilfreichen Vorgehensweisen („best practices“) nicht vorlagen, fielen diese Angebote – auch aufgrund unterschiedlich weit fortgeschrittener Digitalisierungsprozesse in den Einrichtungen – sehr heterogen aus. Hierdurch entstand ein vielfältiger Erfahrungsschatz zur Umsetzbarkeit neuer Angebote und zum Nutzungsverhalten der jeweiligen Zielgruppe der Einrichtung, der im Rahmen der vorliegenden Studie dokumentiert wird. Dabei standen folgende Forschungsfragen im Zentrum:

1. *Perspektive der Fachkräfte:* Wie veränderten sich Strukturen und Prozesse im Arbeitsalltag von Fachkräften familienfördernder Angebote in der Zeit der Coronapandemie? Welche Kommunikationswege in der Arbeit mit Familien wurden beschritten? Wie wurde professioneller Austausch im Team und darüber hinaus gestaltet?
2. *Perspektive der Eltern:* Welchen Unterstützungsbedarf hatten die Familien während der Pandemie? Welche Angebote der Familienförderung waren in welchem Umfang verfügbar und wie wurden sie genutzt? Wie waren die Kommunikationswege im Kontakt und Austausch mit Fachkräften?

Beide Gruppen wurden außerdem zu Möglichkeiten der Verstetigung und ihren Zukunftsperspektiven befragt.

## Methodik

### Studiendesign und Stichprobe

Um dem explorativen Charakter der Studie gerecht zu werden, wurde ein qualitatives Design gewählt. Der Fokusgruppenansatz eignet sich, um erste Erfahrungswerte in Bezug auf ein noch relativ unbekanntes Phänomen zu sammeln und neue Ideen zu generieren [3]. Die Diskussionsgruppen setzen sich aus Expert:innen zusammen, deren Expertise auf den eigenen Erfahrungswerten und der Auseinandersetzung mit einem Sachverhalt beruht [9]. Teilnehmende Fachkräfte wurden in Zusammenarbeit mit der Auftraggeberin der Studie, der Hamburger Sozialbehörde, rekrutiert. Teilnehmende Eltern wurden über familienfördernde Einrichtungen auf die Studie aufmerksam gemacht. Sieben 1,5- bis 2‑stündige Fokusgruppen mit Eltern und Fachkräften wurden im November und Dezember 2020 durchgeführt und von den Autorinnen anhand eines Gesprächsleitfadens entlang der Forschungsfragen moderiert. Dieser war auf Eltern bzw. Fachkräfte zugeschnitten. Expert:innen wurden im Vorfeld aufgefordert, sich wenn möglich zu diesen Themen auch mit Kolleg:innen auszutauschen, um diese zusätzlichen Erfahrungswerte mit in die Diskussionsrunden einfließen zu lassen.

Die *vier Fachkräftegruppen* setzten sich aus insgesamt 21 Fachkräften 20 unterschiedlicher Einrichtungen der Familienförderung der Stadt Hamburg zusammen und bestanden aus jeweils 4–7 Expert:innen aus jeweils einem Einrichtungsformat:

- Elternschulen (6 Fachkräfte),
- Familienteams (4 Fachkräfte),
- Elternlotsenprojekte (4 Fachkräfte),
- Erziehungsberatungsstellen (7 Fachkräfte).

In *zwei Fokusgruppen* wurden außerdem elf Elternteile befragt, die unterschiedliche Angebote der familienfördernden Einrichtungen nutzten. Zusätzlich wurde *eine Fokusgruppe* mit Elternlots:innen durchgeführt, die die Sicht von durch sie begleiteten Eltern vertraten, deren Muttersprache nicht Deutsch ist. Alle Teilnehmenden wurden vor Beginn der Fokusgruppen vollumfänglich aufgeklärt und unterzeichneten schriftliche Einwilligungserklärungen zur Studienteilnahme und zur Aufzeichnung der Fokusgruppen.

### Auswertung

Zu Auswertungszwecken wurden die Fokusgruppen in Bild und Ton aufgezeichnet. Gesprächsaufzeichnungen wurden nach Abschluss der Gespräche mithilfe einer Transkriptionssoftware [4] inhaltlich-semantisch verschriftlicht. Transkripte der Gesprächsbeiträge wurden anschließend nach der Methode der qualitativen Inhaltsanalyse [3] inhaltlich strukturiert und nach thematischer Relevanz in Bezug auf die zentralen Forschungsfragen zusammengefasst.

## Ergebnisse

### Auswirkung der Pandemie auf Familien

*Fachkräftesicht:* Die meisten Familien (v. a. die Mütter) wirken auf die Fachkräfte *gestresst und überlastet*. Es wurden Ängste und Unsicherheiten wegen der Pandemie, finanzielle Not, Raumenge, innerfamiliäre Konflikte aber auch Scham wegen der eigenen Notlage und fehlende Alltagsstruktur bei den Familien wahrgenommen. Die Kinder zeigten erhöhten Medienkonsum. Die Eltern äußerten den Wunsch nach persönlichen Beratungsgelegenheiten, Austauschmöglichkeiten mit anderen und aufsuchender Arbeit. Durch die veränderte Angebotsstruktur und fehlenden Zugang zu digitalen Angeboten nutzen die Eltern andererseits Angebote oft weniger. Als *positive Folgen* der Pandemie nahmen die Fachkräfte intensivere Zeit mancher Eltern für die Kinder/Familienzeit und eine größere Verfügbarkeit der Väter wahr. Sowohl das Ausmaß der Probleme (v. a. mit Homeschooling, aber auch Belastungen durch finanzielle Not) als auch der Zugang zu digitalen Angeboten ist stark *bildungs- und ressourcenabhängig*. Fehlende Endgeräte bzw. fehlende Internetzugänge wurden in fast allen Fachkräftegruppen als Herausforderung für das Erreichen sozial benachteiligter Familien durch digitale Angebote thematisiert. Vor allem Wohnunterkünfte waren quasi abgeschnitten, weil dort kein WLAN vorhanden war. Durch die fehlende technische Ausstattung kam es zu Schwierigkeiten beim Homeschooling und zu Leistungsdefiziten bei Schüler:innen. Längere Schulschließungen gefährdeten v. a. Kinder in Problemlagen im Hinblick auf die Essensversorgung und auf den fehlenden Blick von außen auf mögliche Gefährdungssituationen.

*Elternsicht:* Am häufigsten wurden psychische und physische Belastungen der Eltern, genannt, insbesondere extreme Erschöpfung aufgrund von Mehrarbeit und verringerten Erholungsmöglichkeiten, teilweise auch gestiegene Ängste und Gefühle der Isolation. Seltener nannten Eltern psychische Veränderungen der Kinder wie z. B. Antriebslosigkeit oder erhöhte Ängstlichkeit. Spannungen in der Familie trugen aus Sicht der Eltern zu diesen Belastungen bei, außerdem partnerschaftliche Konflikte und fehlende Sozialkontakte für Kinder und Eltern. Auch die Eltern beschreiben als positive Auswirkung am häufigsten „mehr Familienzeit“, seltener auch Entschleunigung des Alltags, eine neue Wertschätzung für bestehende Sozialkontakte und Aktivitäten draußen.

### Auswirkung der Pandemie auf Einrichtungen und Fachkräfte

Das offene, niedrigschwellige Konzept war während der Maßnahmen zur Eindämmung der Coronapandemie schwer aufrechtzuerhalten. Besonders der Anmeldezwang und verkleinerte Gruppengrößen wirkten hier einschränkend. Informelle „Tür- und Angelgespräche“ waren über digitale Angebote nicht abzudecken, wodurch die Erreichbarkeit vieler Adressat:innen sank. Bei den nachgefragten Beratungsthemen wurde eine Verschiebung wahrgenommen, es entstand ein höherer Bedarf an Sozial- und Paarberatung, Pandemieaufklärung und Beratung zur Konfliktbewältigung. Dieser Bedarf ließ sich digital nur teilweise decken. Vor allem ängstliche Menschen blieben fern und waren schwer zu erreichen. Ein verstärkter Organisations- und Kommunikationsaufwand durch sich wandelnde Anmelde- und Hygienevorschriften machte sich auf Leitungs- und Fachkräfteebene bemerkbar. Hinzu kamen eigene Belastungen bei den Fachkräften durch die Pandemie, Sorge um die betreuten Familien und eine Verunsicherung bezogen auf die Grenzen der Nutzung digitaler Kontaktformen, sowie auf das eigene Gesundheitsrisiko bei Präsenzangeboten. Das veränderte Arbeitsumfeld wurde differenziert wahrgenommen, so bietet das „Homeoffice“ mehr Flexibilität, jedoch auch eine Vermischung von Privatleben und Beruf. Die Nutzung neuer digitaler Medien war herausfordernd, die Kanalverflachung durch Videoformate einschränkend für die Kommunikation.

### Erfahrungen mit neuen Angebotsformen

*Nicht-digitale Angebote* wurden, soweit es ging, nach draußen verlagert: Spaziergänge, Essensausgabe, Spieleausgabe, Spielplatztreffen, im Sommer auch Outdoor-Gruppentreffen. Es entstanden neue nicht-digitale Wege der Zielgruppenansprache und -information, z. B. durch Werbung im Wochenblatt oder den Flyer im Briefkasten. Hinzukamen neue Angebote wie Mittagspakete und Lebensmittelgutscheine, Ausflüge in den Sommerferien, neue Gruppenkonzepte und Angebote angepasst an aktuelle Bedarfe (z. B. Konfliktlösungstraining, Bastelideen). Bewegungsräume wurden den Familien, sobald es ging, als Treffpunktmöglichkeit zur Verfügung gestellt. Manche Einrichtungen arbeiten weiter aufsuchend, andere nicht, um Fachkräfte zu schützen. *Neue digitale Angebote* entstanden in dieser Zeit. Information und Austausch erfolgte über Messengerdienste und Videochat, die Website wurde als Informationsdienst intensiver genutzt. Es entstanden Onlinekurse, wie Rückbildung, Yoga, Babymassage, Elterntreff/Erzählcafé, Bastelangebote für Kinder und eine Online-Musikstunde. Es wurden Tipps für digitale Beschäftigungsmöglichkeiten (wie z. B. Bastel- und Beschäftigungs-Apps, Videos etc.) zur Alltagsentlastung der Familien verschickt.

*Nutzungserfahrungen aus Sicht der Fachkräfte:* Messengerdienste wie WhatsApp bewährten sich für schnellen Austausch und Informationen über aktuelle Angebote (z. T. mehr als die Website), aber auch für die Erreichung der Zielgruppe der Väter. Weniger bekannte Messengerdienste wurden von den Familien nicht gut angenommen, was für Datenschutz als problematisch bewertet wurde. Auf Onlinekurse und Gruppenangebote gab es vielfach positive Resonanz. Durch kleinere Gruppengrößen gab es häufig einen intensiveren Kontakt mit den Familien als vorher, z. T. auch durch thematische Verschiebung. Dies berichteten auch die Elternlotsen: Durch die reduzierte Bürokratie in der Pandemie war mehr Raum für persönliches Gespräch. Digitale Beratung/Sprechstunden und Social-Media-Kommunikation wurden nur z. T. angenommen. Manche Einrichtungen beschrieben auch eine digitale „Müdigkeit“ der Familien aufgrund von Homeschooling. Die Nutzung digitaler Angebote hing von der Ausstattung der Familien, aber auch von der Einrichtungsform ab. In Elternschulen, die vorher einfach aufgesucht wurden, wurde Telefonberatung wenig genutzt. Besonders bei Elternlotsen und Familienbildungsstätten war die Notwendigkeit, sich vorher anzumelden, eine Hürde. Belastete Familien, die wegblieben, mussten aktiv und häufig kontaktiert werden, um das Wohlergehen der Kinder sicherzustellen. In Erziehungsberatungsstellen wurde Onlineberatung gut genutzt und die Fachkräfte erlebten auch unerwartete positive Effekte. So lief die Beratung mit hochstrittigen Eltern in Trennung besser als in Präsenz.

*Nutzungserfahrungen aus Sicht der Eltern:* Die Eltern schätzten am meisten die Aufrechterhaltung des Kontakts egal in welcher Form, und die Möglichkeit, sich in kleineren Gruppen zu treffen, sobald es wieder möglich war. Vermisst wurde der Austausch mit anderen Eltern. Ein Hemmnis für die Nutzung digitaler Angebote war die ohnehin erhöhte Medienzeit der Kinder. In manchen Fällen kam das Fehlen technischer Ausstattung oder die mangelnde Aktualisierung der Angebote seitens der Einrichtung hinzu.

### Veränderte (inter)professionelle Kommunikation

Die Möglichkeit, Teamtreffen, Arbeitskreise und Gremien über Videokonferenz abzuhalten, wurde als bereichernd bewertet, sowohl innerhalb der Einrichtungen, als auch nach außen (z. B. mit Kooperationspartnern). Es wurde aber auch der Wunsch ausgedrückt, wieder mehr zu persönlichen Treffen zurückzukehren. Der Kontakt mit dem Allgemeinen Sozialen Dienst (ASD) und dem Sozialamt wurde regional unterschiedlich bewertet, z. T. wurde die Erreichbarkeit schwieriger. Von der Sozialbehörde hätte man sich mehr spezifische Handlungsanweisungen für die Familienförderung gewünscht, allerdings wurde der daraus entstehende Handlungsspielraum z. T. auch begrüßt.

### Zukunftsperspektiven

*Aus Sicht der Fachkräfte: *Der persönliche Kontakt ist nicht durch digitale Angebote ersetzbar. Auch zukünftig sind online-Angebote wie Beratung oder kreative Ideen zur Entlastung für Familien als ergänzendes Angebot sinnvoll, auch um neue Zielgruppen zu erreichen (z. B. mehr Alleinerziehende). Auch die entstandenen Outdoor-Angebote sollen fortgesetzt werden, ebenso wie die Möglichkeit, sich im Netzwerk per Videokonferenz auszutauschen. Dies schafft eine hohe Verbindlichkeit und Flexibilität, spart aber auch Zeit und Wege. Auch das Feedback zwischen Einrichtungen der Familienförderung und der Sozialbehörde kann dadurch verbessert werden. Für professionelle Onlineangebote brauchen Einrichtungen Ressourcen zur Verbesserung der technischen Ausstattung sowie Fortbildung im Umgang mit digitalen Medien. Während der Pandemie variierte die Qualität letztlich mit der Vorkenntnis der einzelnen Fachkraft.

*Aus Sicht der Eltern:* Auch die Eltern wünschen sich eine (teilweise) Rückkehr zu Präsenzangeboten. Sie vermissten aber ebenfalls spezifischere Angebote, die auch digital sein könnten, für Eltern in unterschiedlichen Lebens- und Bedarfslagen (Kommunikationskurse für Familien, Stressabbau, Umgang mit der Pandemie, Selbstfürsorge, Meditation, Partnerschaft, Umgang mit von Behinderung betroffenen Kindern). Für ihre Kinder wünschen sie sich altersbezogene, kreative Handlungsanregungen (z. B. Experimentiervideos, Buchvorlesungen, Puppentheater, Selbstverteidigung). Insgesamt wurde eine integrierende, übersichtliche Kommunikation über die unterschiedlichen Angebote im Sozialraum angeregt, z. B. eine Website für jeden Stadtteil mit Informationen und Buchungsmöglichkeiten für digitale Angebote.

## Diskussion

Unsere Ergebnisse passen zu einer wachsenden Anzahl von Studien, die psychische Belastungen durch Corona für Fachkräfte der Sozialen Arbeit [6] und für Familien (v. a. mit jungen Kindern und in belasteten Lebensumständen) aufzeigen [1, 10, 11]. Digitalisierung ist notwendig, um auf zukünftige Pandemiesituationen vorbereitet zu sein und jüngere Zielgruppen zu adressieren. Einrichtungen sollten ihre Angebotsstruktur überdenken. Gleichzeitig wurde in der vorliegenden Untersuchung wie auch bei einer Studie der Frühen Hilfen [13] deutlich, dass digitale Angebote weder für Eltern noch für Fachkräfte die Präsenzangebote ersetzen können. Jentsch und Gerber [7] beschreiben ebenfalls ein „mix and match“ digitaler und analoger Mittel in der familienbasierten Arbeit als hilfreich für die Begleitung von Familien durch Fachkräfte der Kinder- und Jugendhilfe. Daraus ergeben sich folgende Handlungsempfehlungen für Einrichtungen der Familienförderung:

- *Bestehende und neue Offlineangebote durch digitale Angebote ergänzen.* Ressourcen- und Berichtsstrukturen anpassen. Kursangebote, Gruppenangebote und Telefonberatung sind für viele Familien passend. Informelle Möglichkeiten des Austauschs entstehen jedoch digital fast gar nicht.
- *Messengerdienste nutzen*, um auf Angebote der Einrichtung, Terminänderungen und freie Plätze in Kursen hinzuweisen.
- *Einrichtungs- oder sozialraumbezogen differenziertere Bedarfsanalysen* durchführen, um Passung von Angebot und Nachfrage sicherzustellen.
- *Neue digitale Angebote im Hinblick auf Handhabbarkeit und Effektivität laufend evaluieren.* Die Technik bietet hier vielfältige Möglichkeiten wie z. B. Onlinefragebögen.

Sowohl zwischen Familien als auch zwischen Fachkräften verlaufen unsichtbare Gräben, was die Nutzung digitaler Möglichkeiten angeht. Der in der Diskussion um das „digital divide“ (die „digitale Spaltung“) stets beschworene Prozentsatz an Personen, die das Internet nicht oder nur widerwillig nutzen wurde bereits 2006 von Riehm und Krings [14] in ihrem Artikel „Abschied vom Internet für alle“ beschrieben. Auch in der vorliegenden Untersuchung zeigt sich, dass es Familien gibt, die online nicht oder nur sehr schwer erreichbar sind. Doch auch gerade bei vielen teilnehmenden Fachkräften zeigte sich eine große Skepsis bezüglich digitaler Angebote, sei es die Frage nach der richtigen Plattform, sei es die Art des Angebots, sei es die technische Ausstattung. Daraus ergeben sich folgende Handlungsempfehlungen:

- *Kein übermäßiger „Digitalisierungsdruck“*, da der Zwang zur Digitalisierung in der Pandemie als belastend erlebt wurde. Offlineangebote sollten weiterhin zentral für die Arbeit in der Familienförderung bleiben.
- Es ist jedoch zu prüfen, inwieweit die Familien aufgrund geringer Motivation diese Angebote nicht nutzen, und an welchen Stellen durch bessere technische Ausstattung und *niedrigschwellige Einweisungen der Familien* eine langfristig höhere Nutzerquote erreicht werden kann.
- *Qualifizierungsmaßnahmen und fortlaufender fachlicher Austausch* der Einrichtungen untereinander. Eine Möglichkeit hierfür wäre die Identifizierung von Multiplikator:innen für digitale Angebote in den Einrichtungen. Multiplikator:innen helfen vor Ort, Hürden gegenüber digitalen Möglichkeiten abzubauen, und können das Thema zentral, z. B. im Rahmen eines Arbeitskreises vorantreiben.

Ein überraschend einheitlich positiver Befund war die *Effektivität von Onlinemeetings* – sei es intern im Team oder extern (vgl. auch [7]). Onlinesitzungen werden als verbindlicher, strukturierter und effektiver erlebt. Somit bietet sich hier das Potenzial, durch digital optimierten Austausch an den Schnittstellen *Ressourcen zu sparen*: viele Akteure können zeitlich flexibel eingebunden werden, Wege entfallen, Sitzungstermine haben eine hohe Verbindlichkeit, weil kaum Hürden zur Teilnahme bestehen, wenn die entsprechende technische Ausstattung vorliegt. Gleichzeitig wünschen sich Eltern mehr differenzierte Vielfalt der Angebote und einen besseren Überblick über Angebotsstrukturen im Stadtteil, was im Sinne von Arbeitsteilung und Ressourcenbündelung eine intensivere Kooperation der Akteure im Sozialraum und darüber hinaus nahelegt. Ein gleitender Übergang zu Präsenzangeboten erfordert die Beibehaltung sozialräumlicher Strukturen, jedoch gibt es auch Themenfelder, für die ein Angebot stadtteilübergreifend erfolgen könnte (z. B. Onlinekurse zur Selbstfürsorge für junge Adoptiveltern und weitere speziellere Themen). Daraus ergeben sich folgende Handlungsempfehlungen:

- *Netzwerktreffen* im Sozialraum z. T. online durchführen,
- *neue Arbeitsstrukturen* für gegenseitige Kooperation bei spezifischen Onlineangeboten schaffen,
- *Bündelung und übersichtliche Darstellung von Onlineangeboten* der Familienförderung im Sozialraum z. B. in Form eines übergreifenden Portals könnte Ziel einer Weiterentwicklung der familienfördernden Angebote werden. Sozialraum übergreifende Onlineangebote von Einrichtungen könnten hier ebenfalls zur Verfügung gestellt werden.

## Limitierung der Studie

Eingeschränkt wird die Interpretierbarkeit der Ergebnisse durch den qualitativen Ansatz und die kleinen Gruppengrößen. Genauere Angaben zur Stichprobe, wie sozioökonomischer Status oder Anzahl der Kinder wurden nicht systematisch erhoben. Eine quantitative Untersuchung sollte unterschiedliche Zielgruppen und soziale Milieus in den Blick nehmen, wie an anderer Stelle auch bereits erfolgte [8, 11]. Zudem mussten die Fokusgruppen, bedingt durch das rasante Pandemiegeschehen, online stattfinden, sodass nur „digital verfügbare“ Familien erreicht werden konnten. Der Zeitpunkt der Durchführung hatte vermutlich großen Einfluss auf die Stimmungslage von Eltern und Fachkräften, sodass eine Follow-up-Befragung mit mehr Abstand zur Pandemie sinnvoll wäre.

## Fazit für die Praxis

- Die Pandemie führte zu vielfältigen Belastungen bei Familien und Fachkräften aus der Familienförderung, aber auch zu neuen Angebotsformen und Erfahrungen mit deren Nutzung.
- Niedrigschwellige, informelle Begegnung und Austausch können nur durch Präsenzangebote beibehalten werden, ansonsten gehen Zielgruppen verloren. Es ist aber sinnvoll, diese durch Onlineangebote zu ergänzen, um spezifischere Bedarfe der Familien zu decken und neue Nutzergruppen zu erreichen.
- Fachkräfte benötigen für die Arbeit mit Onlineangeboten Qualifizierung und Ressourcen, sowie die zentrale Bündelung von zielgruppenspezifischen Angeboten. Ein kontinuierlicher Austausch mit anderen Einrichtungen über die Umsetzung von Onlineangeboten erscheint sinnvoll.
- Interprofessionelle Kooperation profitiert von digitalen Möglichkeiten wie Videoconferencing, weil sie zu einer erhöhten Flexibilität, Verbindlichkeit und Vernetzung beitragen.
